# The Influence of Malarial Poison upon the Upper Abdominal Viscera, and Especially upon the Spleen

**Published:** 1888-08

**Authors:** L. B. Underhill

**Affiliations:** New Braunfels


### THE INFLUENCE OF MALARIAL POISON UPON THE UPPER ABDOMINAL VISCERA, AND ESPECIALLY UPON THE SPLEEN.

By L. B. Underhill, M. D., New Braunfels.

Read at Austin District Medical Society, June 14, 1888, and Published by Vote of Committee.

THE malarial poison is assumed to act on the blood, but the exact mode of production of the lesions, following its intoxicating influence is not, to this date, fully ascertained.

In a discussion of this subject, it will be necessary to speak very briefly concerning the action of the involuntary muscles of organic life.

These involuntary muscular fibres are met with in the uterus, stomach, intestines (large and small), corpora cavernosa of the penis, in the middle coat of the arteries, and especially in the anatomy of the spleen, besides other organs not necessary to mention at present writing. In the larger arteries, the middle, or muscular, coat is either very thin or entirely wanting; their outer, or elastic, coat being very thick and strong, and extremely elastic.

In the smaller vessels, (*i. e.*, arteries,) the middle coat (*vide* before,) predominates in strength and in quantity of tissue, and it possesses great contractility. The splenic frame-work, to which I shall mostly confine my remarks, consists, as you know, of capsule, trabeculæ and ensheathing canals, in which all the vessels lie. The capsule is an elastic tunic, in which are found pale, involuntary muscular fibres. This capsular coat is reflected into the body of the spleen, and canaliculæ, or canal enclosing the vessels (both), and prolongs itself so as to be distributed through the organ, enclosing alike vessels and pulp substance. The large vessels lie enclosed in this contractile tissue, and although they of themselves have but little contractile power, their calibre is regulated by the ensheathing canals enclosing them. The smaller vessels have in themselves contractile power.

These unstriped muscular fibres ramifying throughout the spleen are controlled by the sympathetic or ganglionic system of nerves. These ganglia also receive fibres from the cerebral and spinal nerves, and thus we see that the cerebro-spinal system also influences the unstriped muscular fibres, as they inhibit the influence of the sympathetic. (*Vide* Fotherfill and others.) The action of the sympathetic nerve is to keep up contraction of the unstriped muscular fibres all through the body, the application of cold or electricity to the sympathetic causes contraction of the small vessels or any other structures containing unstriped mus cular fibres.

Increased cerebro-spinal nerve action paralyzes almost entirely the sympathetic nerve fibres and regulates the degree of its influence, and when extreme, causes relaxation by its inhibiting action on unstriped muscular fibres. Withdraw the influence of the cerebro-spinal nerves and we have excessive action of the sympathetic. When the action of the sympathetic on the middle coat of the small arteries is inhibited by any influence of the cerebro-spinal nerve, it becomes relaxed,—self-evident.

The circulating fluid, blood, exerts a certain amount of pressure on the blood vessels; and when the arterial muscular fibres are relaxed, there is little opposition to this pressure, and we have an increased supply of blood to the part, and a general hyperæmia of all its small vessels. This inhibitory action of either the cerebral or spinal nerves is proved by many examples, viz: nervousness, emotion, etc., causes inhibition of the sympathetic nerves supplying the vessels of the head and neck, dilatation or hyperæmia ensue and causes blushing. Under the influence of terror there is temporary paralysis of the cerebro-spinal nerves, the sympathetic has no antagonism, and we have a blanched and pale face.

Erotic thoughts causes hyperæmia of the corpora cavernosa. Thoughts of certain tasty dishes cause hyperæmia of the vessels of the salivary glands, and we have an increased secretion of saliva, and the mouth waters, so to speak.

In the spleen the involuntary muscular fibres are governed in the like manner by the sympathetic, and are influenced by the cerebro-spinal nerves.

The changes which take place during digestion in the stomach, liver and especially the spleen, are similar to those which take place during the temporary influence of a paroxysm of malarial fever, and these changes are mentally caused by nervous influence. The spleen, however, is acted upon toward the end of the process of digestion, when the newly assimilated pabula have entered the blood, and it is from the action of the pabula upon the nerves that we have its temporary enlargement by causing dilation of its vessels. After the whole process of digestion is completed, the sympathetic nerve begins to assert its functions and the temporary engorgement ceases.

There are conditions which react on the sympathetic, causing inhibition of its action locally, and thus a dilation of the vessels of that special part.

We have seen that the emotions, acting through the cerebro-spinal system, cause local inhibition of the sympathetic, and that a suspension of its action causes the reverse. The reason why this is a fact we do not know, but it must be conceded that the cerebro-spinal system of nerves may, or it possibly, can cause only a local action on a certain particular region or organ.

Now, certain substances introduced into the blood have the power of inibiting or stimulating the action of the sympathetic on the blood vessels locally, or on the involuntary muscular fibres in other structures. Strychnia causes dilation of the vessels of the cord. Belladonna causes contraction of the arteries of the cord. Ergot has the same effect, and also contracts the muscular fibres of the gravid uterus.

These different actions are exerted through the spinal nerves, influencing, locally, the sympathetic, and through them the involuntary fibres of different structures.

It is thus not astonishing that the presence of the malarial poison in the blood is capable of exerting a powerful influence upon the vascular system of the stomach, liver and spleen. It seems almost certain that the malarial poison has the effect of dilating the vessels of the spleen and that its mode of action is an influence exerted upon the cerebro-spinal nerve fillaments, existing in the sympathetic ganglia, and as we have remarked before in the case of strychnia and belladonna, the emotions, etc., and that this action is a local one and especially directed upon the involuntary muscular fibres of the upper digestive viscera.

The persistent and obstinate irritability of the stomach and liver during malarial attacks goes clearly to prove that these organs, also, are affected by the poison. P. M. appearances help us to hold our ground.

Why the action of the malarial poison is directed, especially locally, against the digestive viscera, we cannot explain any more than we can explain the local action of the emotions, belladonna, or ergot, but the fact to us seems clear that the malarial poison is essentially a nervine poison.

Its local action most probably takes place through the ganglia themselves, the stimulus being produced in the ganglionic fibres of the spinal system—by them conveyed to the ganglia and exerting there the inhibitory effect on the sympathetic, causing relaxation, dilation and hyperæmia.

The malarial poison acts upon the digestive viscera with unvarying certainty and insidiousness, whether or no, the patient notices the usual febrile symptoms.

The most intense hyperæmia of the spleen and liver occurs during the cold stage of a malaria paroxysm, the irritabilty of the stomach occurring later, and we are very prone to consider that the condition of the skin in the cold stage is secondary to the action of the poison on the digestive viscera. When the power of the poison on these viscera is exhausted, then the hyperæmia ceases and we note a rush of blood to the cutaneous tissues, i. e. the hot stage. The exaggerated action of the sudatory glands soon relieves this hyperæmia, and we have the sweating stage, decreased temperature and final end of the paroxysm.

